# Alkyl Nitrite (“Poppers”) Exposures in the US

**DOI:** 10.1001/jamanetworkopen.2025.23408

**Published:** 2025-07-28

**Authors:** Samantha Kerester, Joshua Bloom, Lauren Schwartz, Maria Mercurio-Zappala, Joseph J. Palamar, Mark K. Su

**Affiliations:** 1 Ronald O. Perelman Department of Emergency Medicine, NYU Grossman School of Medicine, New York, New York; 2 New York City Health and Hospitals Jacobi/North Central Bronx, Bronx, New York; 3 New York City Poison Center, New York City Department of Health and Mental Hygiene, New York, New York; 4 Department of Population Health, NYU Grossman School of Medicine, New York, New York

## Abstract

This case series examines poison center reports of alkyl nitrite (ie, poppers) use throughout an 11-year period.

## Introduction

Alkyl nitrites, or poppers, are drugs that are commonly used for their short-acting vasodilatory properties and purported ability to enhance sexual intercourse.^1^ Whereas inhalation is the typical route of administration, ingestion has recently become more common, possibly because of packaging that closely resembles energy shot beverage products.^1^ Poppers use can cause methemoglobinemia, a potentially life-threatening condition characterized by impaired ability of hemoglobin to carry oxygen to tissues.^2,3,4^ This study analyzed poison center reports during an 11-year period, and we hypothesized that the number of poppers exposures increased during this period and that ingestion caused more severe outcomes than inhalation exposures.

## Methods

This case series was reviewed and approved by the New York City Department of Health and Mental Hygiene institutional review board. Informed consent was not obtained because data were deidentified and collected during routine care provided by the poison center. Poison center data collected during 2013 to 2023 by America’s Poison Centers’ National Poison Data System (NPDS) were retrospectively reviewed and cases were deemed eligible if the reported exposure was amyl or butyl nitrite. Joinpoint version 5.0.1 (National Cancer Institute) was used to examine annual trends in the total number of cases, and the percentage of cases with severe clinical outcomes (major, ie, life-threatening, or death). Analyses focused on overall changes between 2013 and 2023 (no joinpoints specified) based on the average annual percentage change (AAPC). Statistical significance was set at with α = .05, and tests were 2-sided. Data were analyzed from June to November 2024.

## Results

From 2013 to 2023, a total of 2431 cases were identified (regardless of route of administration), including 1110 (45.7%) involving inhalation (117 females [10.5%] and 983 males [88.6%]) and 1367 (56.2%) involving ingestion (271 females [19.8%] and 1089 males [79.7%]) (Table). Ingestion and inhalation cases are nonadditive due to multiple routes of exposure within a case (eg, a person both inhaled and ingested), as well as other forms of exposure (eg, ophthalmic or dermal). The total number of exposures increased over time; specifically, exposures involving inhalation increased from 52 in 2013 to 228 in 2023 (AAPC, 17.2%; 95% CI, 13.5%-22.5%) and exposures involving ingestion increased from 78 in 2013 to 277 in 2023 (AAPC, 16.4%; 95% CI, 12.5%-21.8%). Males accounted for 983 cases (88.6%) involving inhalation and 1089 cases (79.7%) involving ingestion. These distributions remained stable over time. Although inhalation exposures increased at a higher rate than did ingestion exposures, the percentage of severe clinical outcomes increased at a faster rate for ingestions than for inhalations (Figure). Specifically, there were severe clinical outcomes from inhalation exposures in 2 cases (3.8%) in 2013 and 14 cases (6.1%) in 2023, which was not significant; however, ingestion exposures increased from 2 cases (2.6%) to 38 cases (13.7%) (AAPC, 17.3%; 95% CI, 8.7%-26.7%).

**Table.** Characteristics of Exposures to Alkyl Nitrites (ie, Poppers) by Route of Exposure and Year in the US, 2013 to 2023

Characteristic	Inhalation				Ingestion			
	2013-2023, No. (%)	2013, No. (%)	2023, No. (%)	AAPC (95% CI)	2013-2023, No. (%)	2013, No. (%)	2023, No. (%)	AAPC (95% CI)
Overall	1110	52	228	17.2 (13.5 to 22.5)	1367	78	277	16.4 (12.5 to 21.8)
Sex								
Female	117 (10.5)	4 (7.7)	23 (10.1)	4.5 (−5.1 to 15.2)	271 (19.8)	19 (24.4)	65 (23.5)	−2.0 (−10.6 to 7.6)
Male	983 (88.6)	48 (92.3)	201 (88.2)	−0.4 (−1.3 to 0.5)	1089 (79.7)	59 (75.6)	207 (74.7)	0.5 (−1.8 to 2.8)
Unknown	10 (0.9)	0	4 (1.8)	NA	7 (0.5)	0	5 (1.8)	NA
Severe clinical outcome^a^								
No	1022 (92.1)	50 (96.2)	214 (93.9)	NA	1225 (89.6)	76 (97.4)	239 (86.3)	NA
Yes	88 (7.9)	2 (3.8)	14 (6.1)	7.7 (−8.6 to 27.3)	142 (10.4)	2 (2.6)	38 (13.7)	17.3 (8.7 to 26.7)

Abbreviations: AAPC, average annual percentage change; NA, not available.

^a^ Major (ie, life-threatening) outcome or death.

**Figure.**
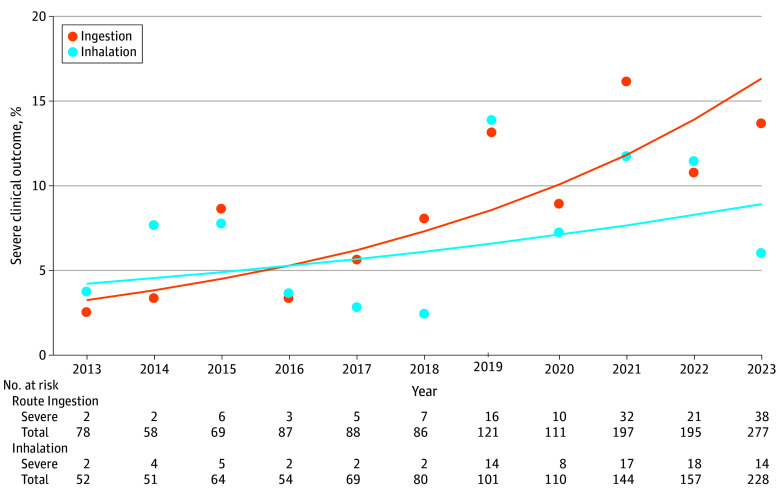
Alkyl Nitrite Severe Clinical Outcomes (Percentage of Total Exposures) by Route of Exposure Dots indicate observed values, and lines indicate fitted values.

## Discussion

These cases demonstrated an increase in alkyl nitrite exposures in the US from 2013 to 2023, with a disproportionate increase in severe clinical outcomes among patients who reported ingestion. High doses of poppers, particularly from ingestion, can cause life-threatening complications, including methemoglobinemia. Limitations of this study include that it is retrospective and is based on voluntary and self-reported aggregate data. Cases with severe clinical outcomes might have been lost to follow-up and not recorded. The data did not allow for subgroup analysis based on presence of methemoglobinemia, which is often not documented in NPDS.

Prevention and harm reduction efforts are needed for those who use poppers. In 2024, the New York City Department of Health and Mental Hygiene began distributing flyers to businesses warning consumers about risks associated with ingestion of poppers. They also released a health advisory^5^ to educate health care clinicians about the diagnosis and management of methemoglobinemia. Public health authorities should both aim to prevent use and advocate for harm reduction practices, such as avoiding ingestion or excessive inhalation. Medical clinicians should be aware of the toxic effects of alkyl nitrites and be able to rapidly diagnose and provide treatment.

## Notes

Supplement.
Data Sharing Statement
