# Supplementary material for: Alkyl Nitrite (“Poppers”) Exposures in the US
Source: JAMA Netw Open. 2025 Jul 28;8(7):e2523408. doi: 10.1001/jamanetworkopen.2025.23408 (PMC12305381; doi:10.1001/jamanetworkopen.2025.23408)
Supplement: Supplement. — Data Sharing Statement [file jamanetwopen-e2523408-s001.pdf]

Data Sharing Statement

Kerester. Alkyl Nitrite (“Poppers”) Exposures in the US. *JAMA Netw Open*. Published July 28, 2025. doi:10.1001/jamanetworkopen.2025.23408

Data
Data available: No

Additional Information
Explanation for why data not available: NA
